# Supplementary material for: Pyruvate Dehydrogenase Kinase 1 inhibition mediated oxidative phosphorylation enhancement in cartilage promotes osteoarthritis progression
Source: BMC Musculoskelet Disord. 2023 Jul 20;24:597. doi: 10.1186/s12891-023-06585-6 (PMC10357736; doi:10.1186/s12891-023-06585-6)
Supplement: Supplementary file 1 — Additional file 1. [file 12891_2023_6585_MOESM1_ESM.zip › MF.Gsea.1653627316242/GOMF_NAD_BINDING.html]

Details for gene set GOMF\_NAD\_BINDING[GSEA]

|  || Dataset | 55457\_collapsed\_to\_symbols.cls.cls #OA\_versus\_normal.cls.cls #OA\_versus\_normal\_repos |
| Phenotype | cls.cls#OA\_versus\_normal\_repos |
| Upregulated in class | OA |
| GeneSet | GOMF\_NAD\_BINDING |
| Enrichment Score (ES) | 0.4141682 |
| Normalized Enrichment Score (NES) | 1.6803954 |
| Nominal p-value | 0.0023094688 |
| FDR q-value | 0.09922041 |
| FWER p-Value | 0.895 |
Table: GSEA Results Summary

  

Fig 1: Enrichment plot: GOMF\_NAD\_BINDING      
 Profile of the Running ES Score & Positions of GeneSet Members on the Rank Ordered List

  

| SYMBOL | TITLE | RANK IN GENE LIST | RANK METRIC SCORE | RUNNING ES | CORE ENRICHMENT || 1 | UXS1 | UDP-glucuronate decarboxylase 1 [Source:HGNC Symbol;Acc:HGNC:17729] | 91 | 0.926 | 0.0549 | Yes |
| 2 | QDPR | quinoid dihydropteridine reductase [Source:HGNC Symbol;Acc:HGNC:9752] | 112 | 0.897 | 0.1133 | Yes |
| 3 | GRHPR | glyoxylate and hydroxypyruvate reductase [Source:HGNC Symbol;Acc:HGNC:4570] | 363 | 0.720 | 0.1420 | Yes |
| 4 | HSD11B2 | hydroxysteroid 11-beta dehydrogenase 2 [Source:HGNC Symbol;Acc:HGNC:5209] | 539 | 0.645 | 0.1715 | Yes |
| 5 | HSD17B8 | hydroxysteroid 17-beta dehydrogenase 8 [Source:HGNC Symbol;Acc:HGNC:3554] | 704 | 0.593 | 0.1984 | Yes |
| 6 | CRYZ | crystallin zeta [Source:HGNC Symbol;Acc:HGNC:2419] | 788 | 0.572 | 0.2302 | Yes |
| 7 | IDH2 | isocitrate dehydrogenase (NADP(+)) 2 [Source:HGNC Symbol;Acc:HGNC:5383] | 967 | 0.525 | 0.2514 | Yes |
| 8 | ALDH2 | aldehyde dehydrogenase 2 family member [Source:HGNC Symbol;Acc:HGNC:404] | 1003 | 0.517 | 0.2833 | Yes |
| 9 | GLUD1 | glutamate dehydrogenase 1 [Source:HGNC Symbol;Acc:HGNC:4335] | 1279 | 0.462 | 0.2928 | Yes |
| 10 | NDUFV1 | NADH:ubiquinone oxidoreductase core subunit V1 [Source:HGNC Symbol;Acc:HGNC:7716] | 1663 | 0.404 | 0.2900 | Yes |
| 11 | ALDH1A1 | aldehyde dehydrogenase 1 family member A1 [Source:HGNC Symbol;Acc:HGNC:402] | 1737 | 0.393 | 0.3106 | Yes |
| 12 | ME1 | malic enzyme 1 [Source:HGNC Symbol;Acc:HGNC:6983] | 1746 | 0.392 | 0.3362 | Yes |
| 13 | SIRT5 | sirtuin 5 [Source:HGNC Symbol;Acc:HGNC:14933] | 1758 | 0.390 | 0.3614 | Yes |
| 14 | ME2 | malic enzyme 2 [Source:HGNC Symbol;Acc:HGNC:6984] | 1870 | 0.377 | 0.3780 | Yes |
| 15 | HPGD | 15-hydroxyprostaglandin dehydrogenase [Source:HGNC Symbol;Acc:HGNC:5154] | 1941 | 0.368 | 0.3971 | Yes |
| 16 | NUDT6 | nudix hydrolase 6 [Source:HGNC Symbol;Acc:HGNC:8053] | 2031 | 0.358 | 0.4142 | Yes |
| 17 | NNT | nicotinamide nucleotide transhydrogenase [Source:HGNC Symbol;Acc:HGNC:7863] | 2425 | 0.311 | 0.4044 | No |
| 18 | RNLS | "renalase, FAD dependent amine oxidase [Source:HGNC Symbol;Acc:HGNC:25641]" | 2955 | 0.254 | 0.3802 | No |
| 19 | NDUFS2 | NADH:ubiquinone oxidoreductase core subunit S2 [Source:HGNC Symbol;Acc:HGNC:7708] | 3072 | 0.243 | 0.3875 | No |
| 20 | CRYL1 | crystallin lambda 1 [Source:HGNC Symbol;Acc:HGNC:18246] | 3292 | 0.221 | 0.3852 | No |
| 21 | PARP1 | poly(ADP-ribose) polymerase 1 [Source:HGNC Symbol;Acc:HGNC:270] | 3410 | 0.210 | 0.3902 | No |
| 22 | GAPDHS | "glyceraldehyde-3-phosphate dehydrogenase, spermatogenic [Source:HGNC Symbol;Acc:HGNC:24864]" | 3466 | 0.205 | 0.3996 | No |
| 23 | ZC3HAV1 | "zinc finger CCCH-type containing, antiviral 1 [Source:HGNC Symbol;Acc:HGNC:23721]" | 3946 | 0.161 | 0.3731 | No |
| 24 | SIRT3 | sirtuin 3 [Source:HGNC Symbol;Acc:HGNC:14931] | 4009 | 0.155 | 0.3787 | No |
| 25 | HADHA | hydroxyacyl-CoA dehydrogenase trifunctional multienzyme complex subunit alpha [Source:HGNC Symbol;Acc:HGNC:4801] | 4163 | 0.141 | 0.3762 | No |
| 26 | SORD | sorbitol dehydrogenase [Source:HGNC Symbol;Acc:HGNC:11184] | 4231 | 0.134 | 0.3800 | No |
| 27 | BDH2 | 3-hydroxybutyrate dehydrogenase 2 [Source:HGNC Symbol;Acc:HGNC:32389] | 4247 | 0.133 | 0.3877 | No |
| 28 | SIRT4 | sirtuin 4 [Source:HGNC Symbol;Acc:HGNC:14932] | 4477 | 0.115 | 0.3776 | No |
| 29 | IDH3G | isocitrate dehydrogenase (NAD(+)) 3 non-catalytic subunit gamma [Source:HGNC Symbol;Acc:HGNC:5386] | 4504 | 0.113 | 0.3831 | No |
| 30 | ME3 | malic enzyme 3 [Source:HGNC Symbol;Acc:HGNC:6985] | 4613 | 0.104 | 0.3816 | No |
| 31 | CTBP2 | C-terminal binding protein 2 [Source:HGNC Symbol;Acc:HGNC:2495] | 5066 | 0.067 | 0.3509 | No |
| 32 | HADH | hydroxyacyl-CoA dehydrogenase [Source:HGNC Symbol;Acc:HGNC:4799] | 5088 | 0.065 | 0.3537 | No |
| 33 | GPD1L | glycerol-3-phosphate dehydrogenase 1 like [Source:HGNC Symbol;Acc:HGNC:28956] | 5381 | 0.041 | 0.3337 | No |
| 34 | ALDH1A3 | aldehyde dehydrogenase 1 family member A3 [Source:HGNC Symbol;Acc:HGNC:409] | 5831 | 0.007 | 0.2992 | No |
| 35 | AOX1 | aldehyde oxidase 1 [Source:HGNC Symbol;Acc:HGNC:553] | 6243 | -0.024 | 0.2688 | No |
| 36 | SIRT2 | sirtuin 2 [Source:HGNC Symbol;Acc:HGNC:10886] | 6279 | -0.026 | 0.2678 | No |
| 37 | ALDH1B1 | aldehyde dehydrogenase 1 family member B1 [Source:HGNC Symbol;Acc:HGNC:407] | 6551 | -0.046 | 0.2498 | No |
| 38 | IDH1 | isocitrate dehydrogenase (NADP(+)) 1 [Source:HGNC Symbol;Acc:HGNC:5382] | 7109 | -0.089 | 0.2124 | No |
| 39 | GPD1 | glycerol-3-phosphate dehydrogenase 1 [Source:HGNC Symbol;Acc:HGNC:4455] | 7420 | -0.112 | 0.1958 | No |
| 40 | EHHADH | enoyl-CoA hydratase and 3-hydroxyacyl CoA dehydrogenase [Source:HGNC Symbol;Acc:HGNC:3247] | 7968 | -0.153 | 0.1634 | No |
| 41 | SIRT6 | sirtuin 6 [Source:HGNC Symbol;Acc:HGNC:14934] | 8532 | -0.196 | 0.1327 | No |
| 42 | SIRT7 | sirtuin 7 [Source:HGNC Symbol;Acc:HGNC:14935] | 8733 | -0.211 | 0.1313 | No |
| 43 | SIRT1 | sirtuin 1 [Source:HGNC Symbol;Acc:HGNC:14929] | 8947 | -0.227 | 0.1299 | No |
| 44 | PHGDH | phosphoglycerate dehydrogenase [Source:HGNC Symbol;Acc:HGNC:8923] | 9245 | -0.252 | 0.1237 | No |
| 45 | CTBP1 | C-terminal binding protein 1 [Source:HGNC Symbol;Acc:HGNC:2494] | 10188 | -0.339 | 0.0730 | No |
| 46 | UGDH | UDP-glucose 6-dehydrogenase [Source:HGNC Symbol;Acc:HGNC:12525] | 10279 | -0.349 | 0.0894 | No |
| 47 | IDH3A | isocitrate dehydrogenase (NAD(+)) 3 catalytic subunit alpha [Source:HGNC Symbol;Acc:HGNC:5384] | 10426 | -0.364 | 0.1023 | No |
| 48 | GLYR1 | glyoxylate reductase 1 homolog [Source:HGNC Symbol;Acc:HGNC:24434] | 10807 | -0.406 | 0.0999 | No |
| 49 | GAPDH | glyceraldehyde-3-phosphate dehydrogenase [Source:HGNC Symbol;Acc:HGNC:4141] | 11268 | -0.467 | 0.0954 | No |
| 50 | IDH3B | isocitrate dehydrogenase (NAD(+)) 3 non-catalytic subunit beta [Source:HGNC Symbol;Acc:HGNC:5385] | 11304 | -0.472 | 0.1242 | No |
Table: GSEA details [plain text format]

  

Fig 2: GOMF\_NAD\_BINDING      
 Blue-Pink O' Gram in the Space of the Analyzed GeneSet

  

Fig 3: GOMF\_NAD\_BINDING: Random ES distribution      
 Gene set null distribution of ES for **GOMF\_NAD\_BINDING**
